# Association of night eating habits with metabolic syndrome and its components: a longitudinal study

**DOI:** 10.1186/s12889-018-6262-3

**Published:** 2018-12-11

**Authors:** Junko Yoshida, Eri Eguchi, Kenjiro Nagaoka, Tatsuo Ito, Keiki Ogino

**Affiliations:** 1 Department of Public Health, Okayama University Graduate School of Medicine, Dentistry and Pharmaceutical Sciences, 2-5-1 Shikatacho, Kita-ku, Okayama, 700-8558 Japan; 2 Department of Nutrition, Faculty of Food Culture, Kurashiki Sakuyo University, Okayama, Japan

**Keywords:** Night eating, Metabolic syndrome, Obesity, Abdominal obesity, Dyslipidemia

## Abstract

**Background:**

Night time eating is a risk factor for metabolic syndrome and obesity. The aim of this study was to investigate whether dinner immediately before bed, snacks after dinner, or combinations of both were associated with metabolic syndrome and its components in a large Japanese cohort.

**Methods:**

We enrolled 8153 adults aged 40–54 years who participated in specific medical checkups in an Okayama facility from 2009 to 2010 and from 2013 to 2014. Age-adjusted and multivariable-adjusted odds ratios of metabolic syndrome and its components in participants with both night eating habits for an average of 3.9 years were evaluated. The relative excess risk due to interaction (RERI) was utilized to determine the supra-additive interaction of both eating habits on metabolic syndrome and its components.

**Results:**

The multivariable-adjusted odds ratio for obesity for those with both eating habits compared to those with neither habit was 2.11 (95% confidence interval [CI], 1.42–3.15) for men and 3.02 (95%CI, 1.72–5.29) for women. Both habits had a supra-additive interaction effect on obesity development in women (RERI, 1.67; RERI%, 85.0; *p* = 0.058), although this result was not significant. In women, there was an association between eating habits at night and metabolic syndrome, but in men it was unrelated. Both night eating habits were associated with dyslipidemia in men and women.

**Conclusions:**

These findings suggest the need for intervention and awareness among individuals with night eating habits to mitigate further complications.

## Background

Obesity and metabolic syndrome are associated with cardiovascular diseases, including cerebrovascular disease and ischemic heart disease, as well as type 2 diabetes mellitus [1–4]. The National Health and Nutrition Survey 2015 showed that 54.0% of men and 18.4% of women aged 40–74 years in Japan were strongly suspected of having metabolic or borderline metabolic syndrome, which are important health concerns in Japan because of the large financial burden associated with metabolic syndrome [5]. A medical checkup that specifically targets metabolic syndrome was introduced in April 2008 to prevent lifestyle-related diseases through early screening. People at risk of metabolic syndrome received specific health guidance by public health nurses and registered dietitians, and they reported improved examination values after three years. Medical expenses and lifestyle-related disease rates also decreased.

Eating habits are directly related to obesity. For example, eating until full and eating quickly can cause unhealthy weight gain [6], and skipping breakfast affects both waist circumference and body mass index (BMI) [7]. In a 4-week trial including a total of 36 obese men and women, breakfast skippers showed an increase in serum total cholesterol [8]. In CARDIA (Coronary Artery Risk Development in Young Adults), daily breakfast eaters also had a lower risk of hypertension (HR, 0.74; 95%CI, 0.63–0.86) and metabolic syndrome (HR, 0.63; 95%CI, 0.54–0.75) than infrequent breakfast eaters (0–3 times a week) [9]. Night time eating, in particular, has been identified as a risk factor for metabolic syndrome and obesity. An intervention study of healthy adults in the United States reported that calorie consumption after 8 PM was positively correlated with BMI and revealed that calorie consumption after 8 PM was an independent predictor of high BMI after adjustments for age, sleep duration, and sleep timing [10]. Late-night eating was associated with an OR for obesity of 1.62 (95%CI, 1.10–2.39) compared with no late-night eating among 3610 Swedish men and women [11]. A cross-sectional study among 239 US adults reported that individuals who consumed ≥33% of their total energy intake (TEI) in the evening had twice the risk of being obese (OR, 2.00; 95%CI, 1.03–3.89) compared with individuals who consumed < 33% of their TEI at night [12]. Soga et al. studied 4912 individuals in their 30s who participated in health checkups for young adults and showed that the multivariable-adjusted odds ratio (OR) for metabolic syndrome was approximately twice as high in women who had dinner immediately before bed than in those who did not [13]. Additionally, the combination of late-night eating and skipping breakfast was associated with a greater risk of metabolic syndrome among Japanese adults (*n* = 60,800; age, 20–75 years) [14]. Another intervention study of fourteen healthy subjects in the United States suggested that eating during the biological night time might increase the risk of weight gain and obesity. Subjects lived for ~ 6 days to simulate a daytime work schedule followed by a 3-day night shift schedule. The total daily energy expenditure decreased by ~ 3% on each of the two night shift days, which consisted of day time sleep followed by afternoon and night time wakefulness [15].

Previous large-scale studies examining night eating habits in Japanese population have reported varying results. Soga et al. reported that among 4912 individual, 43.4% males and 15.2% females had a tendency of “dinner immediately before bed”, and 26.0% males and 23.2% females had a tendency for “snacks after dinner” [13]. Ashizawa.et al., on the other hand, analyzed 278,989 people in Chiba prefecture in Japan and reported that 16.2% males and 9.0% females had a tendency for “dinner immediately before bed”, and 5.9% males and 6.9% females had a tendency for “snacks after dinner” [16]. According to Overview of national medical expenses in 2016, diseases related to metabolic syndrome formed 37.8% of all medical expenses [17], suggesting the need for further investigation into this disease. To our knowledge, no large-scale population-based studies assessing the additive effect of both night eating habits (“dinner immediately before bed” and “snacks after dinner”) on the prevalence of metabolic syndrome and its components have been conducted in Japan.

In the present study, we assessed whether the presence of both night eating habits was associated with metabolic syndrome and its components, such as obesity, abdominal obesity, dyslipidemia, impaired blood pressure, and impaired blood glucose.

## Methods

### Subjects

The participants of this study were 17,534 workers and their spouses aged 40–55 years who underwent medical checkups, with a particular focus on metabolic syndrome, at a facility in Okayama between 2009 and 2010 (baseline). Of these, 9198 workers and their spouses participated in the medical follow-up checkups (between 2013 and 2014; 52.5%). We excluded individuals with missing data on “dinner immediately before bed” and “snacks after dinner” (*n* = 1045). Data of 8153 individuals (4875 men and 3278 women) were used in the final analyses (follow-up rate 46.5%). This study protocol was approved by the ethics committee of Okayama University (approval number: 1032) and conducted in accordance with the Declaration of Helsinki. Considering the longitudinal nature of the study and large number of participants, verbal or written consent was not obtained from all participants. The study was advertised on posters in medical examination centers for a certain period of time. Patients were asked to participate either through the website or in person. Their participation in the survey was taken as consent to participate. This protocol was approved by the ethics committee. The data were analyzed anonymously.

### Measurements

Body mass index (BMI), waist circumference, blood pressure, and metabolites were measured once a year during the annual medical check-up at the Junpukai Health Maintenance Center.

Height and body weight were measured, and BMI was calculated as weight (kg)/height (m^2^). Based on the obesity criteria from the World Health Organization [18], obesity is defined as a BMI ≥25 kg/m^2^. Abdominal obesity was defined as a waist circumference at the navel of ≥90 cm for men and ≥80 cm for women. Blood pressure was measured twice in the sitting position using an automated blood pressure monitor (UDEX-TWIN, Well up).

Blood tests were conducted in a fasting state for the majority of subjects and in a non-fasting state for some subjects. Therefore, fasting blood glucose levels were not available for all subjects. In this study, glycated hemoglobin (HbA1c) levels were monitored and set at ≥5.6%, which is the cut-off value defined for providing specific health guidance in Japan [19]. HbA1c levels were measured using high-performance liquid chromatography. High-density lipoprotein (HDL) and low-density lipoprotein (LDL) cholesterol levels were measured using an enzymatic method (direct method), and triglyceride levels were measured using an enzymatic-based colorimetric method.

Based on the information by IDF [20], we defined metabolic syndrome as any case that showed a waist circumference at the navel of ≥90 cm for men and ≥ 80 cm for women in addition to exhibiting ≥2 of the following components: 1) dyslipidemia, with triglyceride level ≥150 mg/dL, 2) dyslipidemia, with HDL cholesterol level <40 mg/dL for men and < 50 mg/dL for women, 3) hypertension with systolic blood pressure ≥130 mmHg and/or diastolic blood pressure ≥85 mmHg, and 4) hyperglycemia, with HbA1c ≥5.6% [National Glycohemoglobin Standardization Program]. Participants receiving medications for dyslipidemia, hypertension, or hyperglycemia were considered to have the respective condition. Japan Diabetes Society HbA1c values were converted to the National Glycohemoglobin Standardization Program HbA1c values using the officially certified formula: HbA1c (NGSP) (%) = 1.02 × JDS (%) + 0.25%.

### Questionnaire survey

We used the self-administered “standard questionnaire” [19] to assess the participants’ dietary habits. Health care providers in Japan have used this questionnaire since 2008 to evaluate patients for metabolic syndrome. The questionnaire was developed based on the conventional National Health and Nutrition Examination Survey and questions specified by the Industrial Safety and Health Act [19, 21]. It included selection/stratification questions (1–3 [medication use], 4–6 [medical history, present illness], and 8 [smoking history]), which are essential for the specific medical checkup [19]. It also included 22 other items such as frequency of exercise, weight gain, dietary habits, alcohol consumption, and sleeping habits. The participants answered “yes” or “no” to questions concerning night eating habits and other lifestyle-related behaviors. The questionnaire items are standard and have been widely used, and previous studies have showed that these lifestyle behaviors are related to the prevalence of metabolic syndrome [13, 16, 22].

### Night eating habits

Night eating habits were defined as “dinner immediately before bed” (dinner within 2 hours of bedtime ≥3 times/week) and “snacks after dinner” (snacks after dinner ≥3 times/week).

### Other lifestyle-related behaviors

Other lifestyle-related behaviors included smoking (current smoking), exercise (“I have engaged in light ≥30-minute exercise more than twice/week for ≥1 year”), physical activity (“I go for a walk or perform an equivalent physical activity for ≥1 hour/day”), skipping breakfast (“I skip breakfast ≥3 times/week”), and alcohol consumption (current alcohol consumption: “<2 *go* [180 mL/*go*]” or “≥2 *go*”, where “*go*” is a traditional Japanese unit of volume measurement, corresponding to 23 g of ethanol). Alcohol consumption of ≥2 *go* was defined as “excessive drinking”.

### Statistical analysis

The age-adjusted means and proportion of BMI, waist circumference, blood pressure, metabolite levels, metabolic syndrome, and lifestyle-related behaviors (smoking, exercise, physical activity, skipping breakfast, and alcohol consumption) at baseline (2009–2010) were calculated on the basis of the presence or absence of “dinner immediately before bed” and “snacks after dinner” and were compared using analysis of covariance (Dunnett’s multiple comparison test). We compared the groups with one or both eating habits against a group without both habits.

Thereafter, the mean values of the BMI, waist circumference, blood pressure, and metabolite levels compared between baseline (2009–2010) and follow-up (2013–2014) using the Wilcoxon signed rank-tests. The prevalence of metabolic syndrome, night eating habits, smoking, physical activity, and alcohol consumption were compared using the McNemar’s test.

Age- and multivariable-adjusted ORs for metabolic syndrome and its components were calculated using logistic regression analysis in 7287 participants (4262 men and 3025 women), after excluding 866 participants with metabolic syndrome, at baseline to assess the relationships between both eating habits at baseline and the prevalence of metabolic syndrome at follow-up (mean follow-up period, 3.9 years). Similarly, logistic regression analyses for risk of abdominal obesity, obesity, hypertension, dyslipidemia (hypertriglyceridemia), low HDL, and hyperglycemia were performed after excluding individuals with the respective condition at baseline.

Multivariable adjustments were conducted for sex, age, BMI, smoking, alcohol consumption, physical activity, breakfast intake, dyslipidemia, hypertension, and hyperglycemia (“present” or “none” for all items). In the case of one, or both, of the above physical activities and exercises, we referred to them broadly as physical activity in logistic regression analysis. After adjusting for these risk factors, we tested effect modification by sex using an interaction term generated by multiplying variables of age, BMI, smoking, alcohol consumption, physical activity, breakfast intake, dyslipidemia, hypertension, and hyperglycemia. Moreover, we determined whether there was an additive interaction of both night eating habits on the development of metabolic syndrome and its components. We calculated the relative excess risk due to interaction (RERI), which is the excess risk as a result of joint exposure, as the odds ratio (“dinner immediately before bed” + “snacks after dinner”) − the odds ratio (“dinner immediately before bed”) − the odds ratio (“snacks after dinner”) + 1 [6]. We further tested the hypothesis of the RERI = 0 using the Z-test and converted the values to *p*-values [23]. RERI scores > 0 suggest a synergism or greater risk due to interaction than the additive effects of each of both factors [24, 25]. Moreover, the percentage RERI (RERI%), defined as the proportion of disease burden caused by the interaction of two factors [6], was calculated using the following formula: (RERI/odds ratio [“dinner immediately before bed” + “snacks after dinner”]) × 100.

A significance level of 0.05 (two-tailed) was used for all statistical analyses. The SAS statistical software ver. 9.4 (SAS Institute Inc., Cary, NC, USA) was used to conduct all statistical analyses.

## Results

### Participant characteristics

Table 1 shows the mean age, age-adjusted mean values (standard errors), and proportion of baseline characteristics according to the presence of both night eating habits. Compared to participants with neither habit, participants with both habits had a higher BMI, waist circumference, and LDL cholesterol level (*p* < 0.0001 for all). Moreover, a higher proportion of participants with both habits reported skipping breakfast ≥3 times/week.

**Table 1:** Age-adjusted mean values and baseline characteristics according to night eating habits at baseline (2009–2010). Study undertaken in Okayama

	Total	Those with neither habit	Those with a single habit of “dinner immediately before bed” (≥3 times per week)	Those with a single habit of “snacks after dinner” (≥3 times per week)	Those with both habits
Total					
Number of participants (n)	8153	4462	2006	972	713
Mean (SD) Age (years)	46.8	47.0 (0.1)	46.3 (0.1) ^*^	47.1 (0.2)	46.5 (0.2) ^*^
BMI (kg/m^2^)	22.9	22.7 (0.0)	22.9 (0.1)	23.1 (0.1) ^*^	23.9 (0.1) ^*^
Waist circumference (cm)	81.7	81.2 (0.1)	81.6 (0.2)	81.9 (0.3)	84.2 (0.3) ^*^
Systolic blood pressure (mmHg)	117.6	117.8 (0.2)	117.5 (0.3)	116.8 (0.5)	117.2 (0.6)
Diastolic blood pressure (mmHg)	75.3	75.3 (0.2)	75.6 (0.3)	74.8 (0.4)	75.0 (0.4)
Triglycerides (mg/dl)	111.6	111.5 (1.2)	111.2 (1.8)	109.6 (2.6)	115.6 (3.0)
HDL cholesterol (mg/dl)	64.8	64.7 (0.2)	65.5 (0.3)	64.4 (0.5)	63.9 (0.6)
LDL cholesterol (mg/dl)	128.1	127.0 (0.5)	127.9 (0.7)	129.8 (1.0) ^*^	133.6 (1.2) ^*^
HbA1c (NGSP values) (%)	5.54	5.52 (0.01)	5.56 (0.01)	5.57(0.02)	5.56 (0.02)
Metabolic syndrome n (%)	866 (10.6)	399 (8.9)	248 (12.4%) ^*^	113 (11.6) ^*^	106 (14.9) ^*^
Current smokers n (%) ^a^	2134 (28.1)	1022 (24.7)	732 (38.8%) ^*^	173 (19.3)	207 (31.0)
“I have been engaged in light ≥30-min exercise more than twice a week for > 1 year” (%) ^a^	1603 (21.1)	908 (22.0)	381 (20.2%) ^*^	186 (20.7)	128 (19.2)
“Physical activity for > 1 h/day” n (%) ^a^	1258 (16.6)	693 (16.8)	311 (16.5%)	151 (16.8)	103 (15.4)
“I skip breakfast > 3 times a week.” n (%) ^a^	1219 (16.1)	486 (11.8)	448 (23.7%) ^*^	107 (11.9)	178 (26.7) ^*^
Current drinkers n (%)	4582 (56.2)	2360 (52.9)	1429 (71.2%) ^*^	398 (41.0) ^*^	395 (55.4)
Excessive drinkers n (%)	968 (11.9)	438 (9.8)	364 (18.2%) ^*^	64 (6.6)	102 (14.3)
Male					
Number of participants (n)	4875	2357	1619	403	496
Mean (SD) Age (years)	46.9	47.2 (0.1)	46.5 (0.1) ^*^	47.0 (0.2)	46.5 (0.2) ^*^
BMI (kg/m^2^)	23.7	23.6 (0.1)	23.5 (0.1)	24.0 (0.2)	24.6 (0.1) ^*^
Waist circumference (cm)	84.5	84.1 (0.2)	84.3 (0.2)	85.2 (0.4)	86.9 (0.4) ^*^
Systolic blood pressure (mmHg)	120.5	121.0 (0.3)	120.4 (0.4)	119.4(0.7)	119.7(0.7)
Diastolic blood pressure (mmHg)	77.2	77.4 (0.2)	77.4 (0.3)	76.0 (0.6)	76.5 (0.5)
Triglycerides (mg/dl)	133.0	133.6 (2.0)	131.6 (2.4)	129.8 (4.9)	137.4 (4.4)
HDL cholesterol (mg/dl)	59.4	59.3 (0.3)	60.3 (0.4)	57.8 (0.7)	58.4 (0.7)
LDL cholesterol (mg/dl)	131.6	130.2 (0.7)	131.3 (0.8)	135.6 (1.6) ^*^	136.3 (1.4) ^*^
HbA1c (NGSP values) (%)	5.58	5.55 (0.01)	5.59 (0.02)	5.63 (0.03)	5.60 (0.03)
Metabolic syndrome n (%)	613 (12.6)	259 (11.0)	207 (12.8)	63 (15.6) ^*^	84 (16.9) ^*^
Current smokers n (%) ^b^	1869 (41.2)	869 (39.7)	684 (45.0) ^*^	138 (37.5)	178 (38.7)
“I have been engaged in light ≥30-min exercise more than twice a week for > 1 year” n (%)^b^	1097 (24.2)	585 (26.7)	331 (21.8) ^*^	83 (22.6)	98 (21.3)
“Physical activity for > 1 h/day” n (%)^b^	820 (18.1)	411 (18.8)	268 (17.6)	69 (18.8)	72 (15.7)
“I skip breakfast > 3 times a week.” n (%)^b^	920 (20.3)	337 (15.4)	378 (24.9) ^*^	66 (17.9)	139 (30.2) ^*^
Current drinkers n (%)	3368 (69.1)	1605 (68.1)	1226 (75.7) ^*^	226 (56.1) ^*^	311 (62.7)
Excessive drinkers n (%)	878 (18.0)	388 (16.5)	345 (21.3) ^*^	53 (13.2)	92 (18.6)
Female					
Number of participants (n)	3278	2105	387	569	217
Mean (SD) Age (years)	46.7	46.7 (0.1)	45.9 (0.2) ^*^	47.0 (0.2)	46.5 (0.3)
BMI (kg/m^2^)	21.7	21.5 (0.1)	22.1 (0.2) ^*^	21.8 (0.1)	22.9 (0.2) ^*^
Waist circumference (cm)	77.4	76.9 (0.2)	78.0 (0.5)	77.4 (0.4)	80.4 (0.6) ^*^
Systolic blood pressure (mmHg)	113.1	113.1 (0.3)	113.4 (0.8)	112.7 (0.6)	113.7 (1.0)
Diastolic blood pressure (mmHg)	72.5	72.4 (0.2)	72.8 (0.5)	72.5 (0.4)	72.9 (0.7)
Triglycerides (mg/dl)	79.7	79.0 (0.9)	83.1 (2.1)	78.6 (1.7)	82.7 (2.8)
HDL cholesterol (mg/dl)	72.8	72.8 (0.3)	72.5 (0.8)	73.3 (0.7)	72.3 (1.1)
LDL cholesterol (mg/dl)	122.8	122.2 (0.6)	122.6 (1.5)	122.8 (1.2)	129.8 (2.0) ^*^
HbA1c (NGSP values) (%)	5.48	5.47 (0.01)	5.51 (0.02)	5.49 (0.02)	5.49 (0.03)
Metabolic syndrome n (%)	253 (7.7)	140 (6.7)	41 (10.6) ^*^	50 (8.8)	22 (10.1)
Current smokers n (%)^c^	265 (8.7)	153 (7.9)	48 (13.1) ^*^	35 (6.6)	29 (13.9) ^*^
“I have been engaged in light ≥30-min exercise more than twice a week for > 1 year” n (%)^c^	506 (16.6)	323 (16.6)	50 (13.6)	103 (19.5)	30 (14.4)
“Physical activity for > 1 h/day” n (%)^c^	438 (14.4)	282 (14.5)	43 (11.7)	82 (15.5)	31 (14.9)
“I skip breakfast > 3 times a week.” n (%)^c^	299 (9.8%)	149 (7.7)	70 (19.1) ^*^	41 (7.8)	39 (18.8) ^*^
Current drinkers n (%)	1214 (37.0)	755 (35.9)	203 (52.5) ^*^	172 (30.2) ^*^	84 (38.7)
Excessive drinkers n (%)	90 (2.8)	50 (2.4)	19 (4.9) ^*^	11 (1.9)	10 (4.6)

^*^p < 0.05 Comparison of the group with a single habit of either “dinner immediately before bed” or “snacks after dinner”, or the group with both habits, to the group without both habits. *BMI*, body mass index; *HDL*, high-density lipoprotein; *LDL*, low-density lipoprotein; *HbA1c*, glycated hemoglobin; *NGSP*, National Glycohemglobin Standardization Program

### Metabolic syndrome, lifestyle-related behaviors, and biochemical analysis results

Table 2 describes the rates of metabolic syndrome and lifestyle-related behaviors as well as the mean values (standard deviations) of the biochemical analysis results at baseline and follow-up. In participants, the prevalence of metabolic syndrome was 2.5% higher at follow-up than at baseline (*p* < 0.0001). Mean BMI, waist circumference, systolic blood pressure, diastolic blood pressure, and HbA1c levels were also higher at follow-up than at baseline in participants (*p* < 0.0001 for all). Mean triglyceride and LDL cholesterol levels increased in women and decreased in men (p < 0.0001 for all). Regarding lifestyle-related behaviors, the rates of current smoking (p < 0.0001) and skipping breakfast (*p* = 0.002) decreased in participants, whereas the rates of exercise and physical activity increased (p < 0.0001 for both) over time. The rates of current drinking and excessive drinking increased in men (p < 0.0001 for both) and only the rate of excessive drinking increased in women (p < 0.0001). Although improvements over time were observed for most lifestyle-related behaviors (except for drinking) in participants, no changes were seen for “dinner immediately before bed” (24.6% and 23.9% at baseline and follow-up, respectively; *p* = 0.203) and “snacks after dinner” (11.9% and 11.8% at baseline and follow-up, respectively; *p* = 0.559).

**Table 2:** Rate of metabolic syndrome at baseline (2009–2010) and after an average of 3.9 years. Study undertaken in Okayama

	Total		*p* value	Male		*p* value	Female		*p* value
	2009–2010	2013–2014		2009–2010	2013–2014		2009–2010	2013–2014	
Number of participants (*N*)	8153	8153	–	4875	4875	–	3278	3278	–
Age (years)	46.8 (4.6)	50.6 (4.9)	< 0.0001	46.9 (4.8)	50.7 (4.9)	< 0.0001	46.7 (4.7)	50.5 (4.8)	< 0.0001
BMI (kg/m^2^)	22.9 (3.4)	23.1 (3.5)	< 0.0001	23.7 (3.3)	23.8 (3.3)	< 0.0001	21.7 (3.3)	22.0 (3.5)	< 0.0001
Waist circumference (cm)	81.7 (9.6)	82.3 (9.6)	< 0.0001	84.5 (8.9)	84.9 (9.0)	< 0.0001	77.4 (9.0)	78.5 (9.3)	< 0.0001
Systolic blood pressure (mmHg)	117.6 (15.4)	118.8 (16.6)	< 0.0001	120.5 (15.0)	121.8 (15.7)	< 0.0001	113.1 (15.0)	114.2 (16.7)	< 0.0001
Diastolic blood pressure (mmHg)	75.3 (11.4)	75.9 (11.1)	< 0.0001	77.2 (11.3)	78.1 (10.7)	< 0.0001	72.5 (10.9)	72.8 (11.1)	0.044
Triglycerides (mg/dl)	111.6 (84.2)	109.8 (84.6)	0.004	133.0 (97.6)	127.5 (98.6)	< 0.0001	79.7 (42.0)	83.4 (46.8)	< 0.0001
HDL cholesterol (mg/dl)	64.8 (16.3)	65.0 (17.4)	0.356	59.4 (14.5)	59.4 (15.5)	0.145	72.8 (15.6)	73.5 (16.6)	0.002
LDL cholesterol (mg/dl)	128.1 (31.9)	127.7 (31.3)	< 0.0001	131.6 (32.0)	128.5 (31.1)	< 0.0001	122.8 (30.9)	126.6 (31.6)	< 0.0001
HbA1c (NGSP values) *n* (%)	5.54 (0.6)	5.59 (0.6)	< 0.0001	5.58 (0.7)	5.63 (0.6)	< 0.0001	5.48 (0.4)	5.53 (0.5)	< 0.0001
Metabolic syndrome *n* (%)	866 (10.6%)	1066 (13.1)	< 0.0001	613 (12.6)	702 (14.4)	< 0.0001	253 (7.7)	364 (11.1)	< 0.0001
Current smokers *n* (%)^a^	2134 (28.1)	1760 (23.2)	< 0.0001	1869 (41.2)	1539 (33.9)	< 0.0001	265 (8.7)	221 (7.3)	< 0.0001
“I have been engaged in light ≥30-min exercise more than twice a week for > 1 year” *n* (%)^a^	1603 (21.1)	1847 (24.4)	< 0.0001	1097 (24.2)	1249 (27.5)	< 0.0001	506 (16.6)	598 (19.6)	< 0.0001
“Physical activity for > 1 h/day” *n* (%)^a^	1258 (16.6)	1409 (18.6)	< 0.0001	820 (18.1)	920 (20.3)	0.0007	438 (14.4)	489 (16.1)	0.020
Those with a single habit of “dinner within 2 h of bedtime” (≥3 times per week) *n* (%)	2006 (24.6)	1952 (23.9)	0.203	1619 (33.2)	1543 (31.7)	0.036	387 (11.8)	409 (12.5)	0.320
Those with a single habit of “snacks after dinner” (≥3 times per week) *n* (%)	972 (11.9)	962 (11.8)	0.559	403 (8.3)	401 (8.2)	0.488	569 (17.4)	561 (17.1)	0.888
Those with both habits of “dinner within 2 h of bedtime” and “snacks after dinner” (≥3 times per week) *n* (%)	713 (8.8)	694 (8.5)	0.168	496 (10.2)	489 (10.0)	0.468	217 (6.6)	205 (6.3)	0.213
“I skip breakfast > 3 times a week.” *n* (%)^a^	1219 (16.1)	1134 (15.0)	0.002	920 (20.3)	836 (18.4)	0.0001	299 (9.8)	298 (9.8)	1.000
Current drinkers *n* (%)	4582 (56.2)	4683 (57.4)	0.002	3368 (69.1)	3475 (71.3)	< 0.0001	1214(37.0)	1208 (36.9)	0.817
excessive drinkers *n* (%)	968 (11.9)	1238 (15.2)	< 0.0001	878 (18.0)	1109 (22.8)	< 0.0001	90 (2.8)	129 (3.9)	0.0006

BMI, waste circumference, blood pressure, and biochemical analysis results were compared using the Wilcoxon signed rank-tests. The prevalence of metabolic syndrome, current smoker, physical activity, eating habits, current drinker and excessive drinker were compared using McNemar’s test. *BMI*, body mass index; *HDL*, high-density lipoprotein; *LDL*, low-density lipoprotein; *HbA1c*; glycated hemoglobin; *NGSP*, National Glycohemoglobin Standardization Program

### Relationship between night eating habits and metabolic syndrome

Tables 3 and 4 show the relationships between night eating habits at baseline and metabolic syndrome (and its components) at follow-up. At follow-up, 478 individuals (289 men and 189 women) had developed metabolic syndrome. Women with both night eating habits had higher odds of developing metabolic syndrome than those with neither habit (multivariable-adjusted OR, 1.68; 95% CI, 1.00–2.84). No supra-additive interaction of both habits on the development of metabolic syndrome was observed; moreover, the association between both night eating habits and metabolic syndrome did not vary significantly by sex. In men, we did not find a significant association between night eating habits and metabolic syndrome.

**Table 3:** Relationship between two night eating habits and metabolic syndrome after 3.9 years (2013–2014). Study undertaken in Okayama

	Total			
	Those with neither habit	Those with a single habit of “dinner immediately before bed” (≥3 times per week)	Those with a single habit of “snacks after dinner” (≥3 times per week)	Those with both habits
Metabolic syndrome	*n* = 7287			
Number of participants	249/4063	120/1758	58/859	51/607
Model 1^a^	1.00	1.14 (0.90–1.44)	1.12 (0.83–1.51)	1.43 (1.04–1.96)
Model 2^b^	1.00	1.10 (0.86–1.41)	1.13 (0.83–1.54)	1.33 (0.95–1.86)
Abdominal obesity	*n* = 5780			
Number of participants	342/3212	148/1485	87/652	58/431
Model 1	1.00	1.20 (0.97–1.49)	1.18 (0.91–1.52)	1.52 (1.12–2.07)
Model 2	1.00	1.15 (0.92–1.45)	1.19 (0.91–1.55)	1.41 (1.03–1.95)
Model 3^c^	1.00	1.16 (0.93–1.47)	1.19 (0.91–1.55)	1.42 (1.03–1.97)
Obesity	*n* = 6225			
Number of participants	204/3542	128/1489	51/747	63/447
Model 1	1.00	1.32 (1.04–1.68)	1.30 (0.94–1.79)	2.47 (1.82–3.35)
Model 2	1.00	1.31 (1.01–1.69)	1.32 (0.95–1.84)	2.34 (1.69–3.23)
Model 3^c^	1.00	1.33 (1.02–1.71)	1.32 (0.95–1.84)	2.37 (1.71–3.29)
Hypertension	*n* = 6074			
Number of participants	512/3368	252/1431	103/749	80/526
Model 1	1.00	1.04 (0.87–1.23)	0.95 (0.75–1.19)	0.92 (0.71–1.19)
Model 2	1.00	0.97 (0.81–1.17)	0.95 (0.74–1.21)	0.89 (0.68–1.16)
Model 3^d^	1.00	0.98 (0.82–1.18)	0.92 (0.72–1.17)	0.78 (0.59–1.03)
Dyslipidemia	*n* = 6177			
Number of participants	447/3462	239/1450	96/763	102/502
Model 1	1.00	1.16 (0.97–1.39)	1.04 (0.82–1.31)	1.63 (1.28–2.08)
Model 2	1.00	1.20 (0.99–1.45)	1.07 (0.83–1.37)	1.61 (1.25–2.09)
Model 3^e^	1.00	1.18 (0.97–1.43)	1.03 (0.80–1.33)	1.49 (1.14–1.94)
Hyper triglyceridemia	*n* = 6605			
Number of participants	286/3694	176/1535	64/833	71/543
Model 1	1.00	1.21 (0.98–1.49)	1.11 (0.83–1.47)	1.58 (1.19–2.09)
Model 2	1.00	1.15 (0.92–1.42)	1.16 (0.87–1.56)	1.44 (1.07–1.95)
Model 3^e^	1.00	1.13 (0.91–1.41)	1.11 (0.83–1.49)	1.35 (0.99–1.84)
Low HDL cholesterolemia	*n* = 7750			
Number of participants	125/4246	62/1922	27/917	32/665
Model 1	1.00	1.13 (0.82–1.56)	0.99 (0.65–1.52)	1.70 (1.14–2.53)
Model 2	1.00	1.27 (0.91–1.79)	1.07 (0.69–1.67)	1.63 (1.06–2.51)
Model 3^e^	1.00	1.19 (0.84–1.68)	0.99 (0.64–1.55)	1.36 (0.88–2.11)
Hyperglycemia	*n* = 5717			
Number of participants	509/3194	198/1386	106/669	79/468
Model 1	1.00	0.96 (0.80–1.15)	0.97 (0.77–1.22)	1.16 (0.89–1.51)
Model 2	1.00	0.98 (0.81–1.19)	0.92 (0.72–1.18)	1.13 (0.86–1.49)
Model 3^f^	1.00	0.99 (0.82–1.20)	0.90 (0.70–1.15)	1.06 (0.80–1.40)

Overall analysis was adjusted by sex.

^a^Adjusted by age

^b^Adjusted by age, smoking habit (yes, no, missing), alcohol consumption (yes, no), physical activity (yes, no, missing), and breakfast intake (yes, no, missing)

^c^Adjusted by age, smoking habit, alcohol consumption, physical activity, breakfast intake, hypertension, dyslipidemia, and hyperglycemia

^d^Adjusted by age, smoking habit, alcohol consumption, physical activity, breakfast intake, dyslipidemia, hyperglycemia, and BMI

^e^Adjusted by age, smoking habit, alcohol consumption, physical activity, breakfast intake, hypertension, hyperglycemia, and BMI

^f^Adjusted by age, smoking habit, alcohol consumption, physical activity, breakfast intake, hypertension, dyslipidemia, and BMI

*HDL*, high-density lipoprotein

**Table 4:** Relationship between two night eating habits and metabolic syndrome after 3.9 years (2013–2014) by sex. Study undertaken in Okayama

	Male				Female			
	Those with neither habit	Those with a single habit of “dinner immediately before bed” (≥3 times per week)	Those with a single habit of “snacks after dinner” (≥3 times per week)	Those with both habits	Those with neither habit	Those with a single habit of “dinner immediately before bed” (≥3 times per week)	Those with a single habit of “snacks after dinner” (≥3 times per week)	Those with both habits
Metabolic syndrome	*n* = 4262				*n* = 3025			
Number of participants	131/2098	101/1412	25/340	32/412	118/1965	19/346	33/519	19/195
Model 1^a^	1.00	1.18 (0.90–1.55)	1.19 (0.77–1.86)	1.26 (0.84–1.88)	1.00	0.99 (0.60–1.63)	1.05 (0.70–1.57)	1.72 (1.03–2.86)
Model 2^b^	1.00	1.17 (0.88–1.56)	1.19 (0.75–1.90)	1.13 (0.73–1.74)	1.00	0.87 (0.51–1.50)	1.08 (0.72–1.63)	1.68 (1.00–2.84)
Abdominal obesity	*n* = 3665				*n* = 2115			
Number of participants	133/1822	104/1237	26/287	34/319	209/1390	44/248	61/365	24/112
Model 1	1.00	1.18 (0.90–1.54)	1.26 (0.81–1.95)	1.48 (1.00–2.21)	1.00	1.24 (0.87–1.77)	1.13 (0.83–1.55)	1.56 (0.97–2.51)
Model 2	1.00	1.22 (0.92–1.62)	1.15 (0.71–1.85)	1.35 (0.88–2.07)	1.00	1.04 (0.70–1.55)	1.20 (0.87–1.65)	1.47 (0.90–2.41)
Model 3^c^	1.00	1.25 (0.94–1.66)	1.13 (0.70–1.84)	1.36 (0.89–2.09)	1.00	1.03 (0.69–1.53)	1.19 (0.87–1.65)	1.47 (0.89–2.40)
Obesity	*n* = 3413				*n* = 2812			
Number of participants	124/1698	110/1162	29/267	43/286	80/1844	18/327	22/480	20/161
Model 1	1.00	1.32 (1.01–1.73)	1.53 (1.00–2.34)	2.19 (1.51–3.18)	1.00	1.31 (0.77–2.21)	1.06 (0.65–1.72)	3.13 (1.86–5.26)
Model 2	1.00	1.32 (0.99–1.76)	1.57 (1.01–2.45)	2.10 (1.41–3.12)	1.00	1.23 (0.70–2.18)	1.08 (0.66–1.78)	2.90 (1.67–5.05)
Model 3^c^	1.00	1.35 (1.01–1.80)	1.60 (1.02–2.49)	2.11 (1.42–3.15)	1.00	1.22 (0.69–2.16)	1.05 (0.64–1.74)	3.02 (1.72–5.29)
Hypertension	*n* = 3353				*n* = 2721			
Number of participants	312/1609	218/1112	55/285	62/347	200/1759	34/319	48/464	18/179
Model 1	1.00	1.05 (0.87–1.28)	1.00 (0.73–1.38)	0.93 (0.69–1.26)	1.00	0.98 (0.66–1.44)	0.89 (0.64–1.24)	0.88 (0.53–1.47)
Model 2	1.00	1.01 (0.82–1.24)	1.00 (0.72–1.41)	0.88 (0.64–1.21)	1.00	0.84 (0.55–1.26)	0.90 (0.64–1.27)	0.91 (0.54–1.52)
Model 3^d^	1.00	1.03 (0.84–1.27)	0.98 (0.69–1.38)	0.80 (0.58–1.11)	1.00	0.81 (0.53–1.23)	0.86 (0.60–1.23)	0.75 (0.44–1.28)
Dyslipidemia	*n* = 3274				*n* = 2903			
Number of participants	260/1575	205/1115	50/268	70/316	187/1887	34/335	46/495	32/186
Model 1	1.00	1.17 (0.95–1.43)	1.17 (0.83–1.63)	1.49 (1.10–2.00)	1.00	1.13 (0.76–1.67)	0.92 (0.65–1.29)	1.94 (1.28–2.94)
Model 2	1.00	1.19 (0.96–1.47)	1.23 (0.87–1.74)	1.50 (1.09–2.06)	1.00	1.23 (0.81–1.85)	0.94 (0.66–1.35)	1.91 (1.23–3.00)
Model 3^e^	1.00	1.19 (0.96–1.47)	1.20 (0.84–1.70)	1.46 (1.06–2.01)	1.00	1.16 (0.76–1.75)	0.92 (0.64–1.33)	1.66 (1.06–2.61)
Hyper triglyceridemia	*n* = 3507				*n* = 3098			
Number of participants	193/1690	156/1178	44/298	59/341	93/2004	20/357	20/535	12/202
Model 1	1.00	1.19 (0.95–1.50)	1.35 (0.95–1.92)	1.65 (1.20–2.27)	1.00	1.29 (0.78–2.12)	0.79 (0.48–1.30)	1.32 (0.71–2.45)
Model 2	1.00	1.13 (0.89–1.43)	1.40 (0.97–2.02)	1.65 (1.18–2.32)	1.00	1.23 (0.73–2.07)	0.86 (0.52–1.42)	0.88 (0.43–1.80)
Model 3^e^	1.00	1.13 (0.89–1.44)	1.36 (0.94–1.97)	1.61 (1.14–2.27)	1.00	1.16 (0.68–1.96)	0.82 (0.49–1.36)	0.76 (0.37–1.56)
Low HDL cholesterolemia	*n* = 4629				*n* = 3121			
Number of participants	62/2231	50/1559	12/377	21/462	63/2015	12/363	15/540	11/203
Model 1	1.00	1.14 (0.78–1.67)	1.15 (0.61–2.15)	1.65 (1.00–2.74)	1.00	1.08 (0.58–2.03)	0.89 (0.50–1.57)	1.77 (0.92–3.42)
Model 2	1.00	1.25 (0.84–1.87)	1.13 (0.58–2.19)	1.43 (0.82–2.50)	1.00	1.32 (0.69–2.54)	1.03 (0.57–1.84)	2.01 (1.02–3.94)
Model 3^e^	1.00	1.19 (0.79–1.78)	1.00 (0.51–1.97)	1.23 (0.70–2.16)	1.00	1.23 (0.64–2.40)	0.97 (0.54–1.75)	1.67 (0.84–3.32)
Hyperglycemia	*n* = 3322				*n* = 2395			
Number of participants	105/1645	69/1103	17/258	23/316	64/1549	9/283	20/411	10/152
Model 1	1.00	1.12 (0.91–1.39)	1.12 (0.77–1.61)	1.08 (0.77–1.52)	1.00	0.58 (0.39–0.87)	0.89 (0.66–1.19)	1.28 (0.85–1.95)
Model 2	1.00	1.13 (0.91–1.42)	1.01 (0.68–1.50)	1.00 (0.70–1.44)	1.00	0.62 (0.41–0.94)	0.87 (0.64–1.19)	1.35 (0.88–2.06)
Model 3^f^	1.00	1.16 (0.93–1.46)	0.99 (0.66–1.47)	0.96 (0.66–1.38)	1.00	0.61 (0.40–0.93)	0.84 (0.61–1.15)	1.25 (0.81–1.94)

^a^Adjusted by age

^b^Adjusted by age, smoking habit, alcohol consumption, physical activity, and breakfast intake

^c^Adjusted by age, smoking habit, alcohol consumption, physical activity, breakfast intake, hypertension, dyslipidemia, and hyperglycemia

^d^Adjusted by age, smoking habit, alcohol consumption, physical activity, breakfast intake, dyslipidemia, hyperglycemia, and BMI

^e^Adjusted by age, smoking habit, alcohol consumption, physical activity, breakfast intake, hypertension, hyperglycemia, and BMI

^f^Adjusted by age, smoking habit, alcohol consumption, physical activity, breakfast intake, hypertension, dyslipidemia, and BMI

*HDL*, high-density lipoprotein

### Relationship between night eating habits and abdominal obesity

Men and women with both night eating habits had higher odds of abdominal obesity than those with neither habit (multivariable-adjusted OR, 1.42; 95% CI, 1.03–1.97). However, the association between both night eating habits and abdominal obesity did not vary significantly by sex.

### Relationship between night eating habits and obesity (body mass index ≥25 kg/m^2^)

When obesity was evaluated using BMI ≥25 kg/m^2^ as the cut-off, we observed that participants with both night eating habits had higher odds of developing obesity than those with neither habit (multivariable-adjusted OR, 2.11; 95% CI, 1.42–3.15 for men and 3.02; 95% CI, 1.72–5.29 for women). The presence of both night eating habits appeared to have a supra-additive interaction of borderline significance for obesity in women (RERI, 1.67; RERI%, 85.0%; *p* = 0.058). In men, each habit (“dinner immediately before bed” and “snacks after dinner”) was associated with increased odds for developing obesity.

### Relationship between night eating habits and hypertension

Participants with both night eating habits had lower odds of developing hypertension than those with neither habit (multivariable-adjusted OR, 0.78; 95% CI, 0.59–1.03). However, this association was not significant.

### Relationship between night eating habits and dyslipidemia

Men with both night eating habits had a greater probability of developing dyslipidemia (hypertriglyceridemia) than those with neither habit (multivariable-adjusted OR, 1.46; 95% CI, 1.06–2.01 and 1.61; 95% CI, 1.14–2.27, respectively). Women with both night eating habits had higher odds of dyslipidemia than those with neither habit (multivariable-adjusted OR, 1.66; 95% CI, 1.06–2.61). The association between both night eating habits and dyslipidemia did not vary significantly by sex.

### Relationship between night eating habits and hyperglycemia

Women reporting only “dinner immediately before bed ≥3 times/week” had lower odds of hyperglycemia than those reporting neither night eating habit.

## Discussion

Participants reporting both night eating habits (“dinner immediately before bed” and “snacks after dinner”) had higher odds for obesity at follow-up than at baseline. Moreover, we detected a supra-additive interaction of borderline significance for obesity in women with both habits. In men and women, having both night eating habits was associated with dyslipidemia. In men, it was associated with hypertriglyceridemia. Furthermore, sex differences did not seem to affect the prevalence of metabolic syndrome and its components, as the tests for effect modification by sex were not significant in those with both night eating habits.

A previous randomized crossover trial that compared the effects of night and day snack intake of approximately 200 kcal daily for 13 days showed that the blood LDL cholesterol level was higher in the night time snack intake group than in the daytime snack intake group [26]. An epidemiological study of 150 male bus drivers indicated that night workers had a higher risk of obesity (BMI ≥25 kg/m^2^) and abdominal obesity (waist circumference ≥94 cm) than daytime workers [27]. Furthermore, having dinner immediately before bed was reported to be significantly associated with metabolic syndrome and hyperglycemia [13, 28], and men who ate snacks after dinner had a higher risk of coronary heart disease than men who did not [29]. A previous study showed an association between “dinner immediately before bed” and metabolic syndrome in women, but not men [13].

In our study, men and women with both night eating habits showed higher ORs for obesity than those without either habit. Noticeably, both habits tended to show a supra-additive interaction for developing obesity in women, although this result was not statistically significant (RERI, 1.67; RERI%, 85.0%; *p* = 0.058). In men, each habit (“dinner immediately before bed” and “snacks after dinner”) was associated with higher odds of developing obesity. Moreover, men with both night eating habits had higher odds for dyslipidemia (hypertriglyceridemia) when compared to those with neither habit.

There are several explanations for the observed association between night eating habits and increased odds for developing metabolic syndrome and its component in women. Late-night eating could lead to circadian misalignment, reduced energy expenditure (reduced levels of leptin), increased appetite sensations, and weight gain [30]. In addition to behavioral chronodisruption, circadian gene variations have been associated with altered metabolism resulting in negative health outcomes. For example, clock gene mutation carriers, in the minor C allele, were resistant to weight loss after a 12–14-week intervention. In addition, they had shorter sleep durations, higher ghrelin concentrations, and reported a preference for delayed breakfast time and evening meals [31]. Energy expenditure is lower at night than during the day [15, 32]. Therefore, when compared to daytime snacking, night time snacking has been associated with decreased fat oxidation [33] and can cause fat accumulation because nutrients ingested at night are not used for glycogen synthesis in muscles and the liver [34]. Shimba et al. reported that the circadian clock gene *BMAL1*, which regulates circadian rhythms and adipogenesis, is primarily activated from 10 PM to 2 AM, and that the overexpression of *BMAL1* in adipocytes increases lipid synthesis activity [35]. Food intake at night might induce the overexpression of *BMAL1* and cause visceral fat accumulation. Additionally, once obesity develops, insulin resistance and metabolic inflammation due to the dysfunction of adipocytokine secretion, which includes increased tumor necrosis factor-α and resistin as well as a decreased adiponectin, are triggered and result in metabolic dysfunction [36]. Finally, postprandial glucose, insulin, and triglyceride levels are significantly elevated when consuming meals at night compared to during the day, resulting in diminished insulin sensitivity [37]. Insulin activates lipoprotein lipase and suppresses the secretion of very low-density lipoprotein (VLDL) cholesterol in the liver. Therefore, lower nocturnal insulin sensitivity could be associated with lower lipoprotein lipase activity and higher circulating plasma triglyceride levels [33]. Therefore, eating at night may lead to metabolic syndrome and its components.

The present study did not observe a significant association between night eating habits and the development of hypertension and hyperglycemia. Nevertheless, “dinner immediately before bed” is an important factor that increases the risk of hypertension and hyperglycemia [28, 38].

Moreover, we found that the prevalence of lifestyle-related behaviors (smoking, skipping breakfast, and physical activity) improved at follow-up when compared to the baseline, presumably due to the provision of specific health guidance. However, no decrease in the rates of “dinner immediately before bed”, “snacks after dinner”, and alcohol consumption was observed. For these reasons, interventions and awareness are important for individuals reporting both night eating habits to help reduce further complications.

### Study limitations and strengths

One limitation of the present study was the low follow-up rate (46.5%). This was due to individuals not undergoing the re-examination at follow-up and/or having missing data on night eating habits in the questionnaire. However, we compared the characteristics of participants and non-participants of the follow-up examination and found no major differences in the prevalence of metabolic syndrome and lifestyle-related behaviors as well as for the results of the biochemical analyses. Thus, the effect of attrition seems to have been weak. The questionnaire included data on eating habits but did not include meal content related questions. Therefore, it is possible that healthy snacks were being consumed at night time. To establish a conclusive relation between night time eating habit and metabolic syndrome, snacking habits of men and women with and without metabolic syndrome should have been monitored. This was not done in the study. Another limitation of this study was that data on sleep duration was not available. Previous studies of healthy participants aged 20–30 years reported that short sleep duration increased energy intake and may contribute to obesity [38–42]. Furthermore, in the present study, some female participants were approaching menopause. Reduced energy expenditure owing to decreased estrogen secretion [43] and deleterious changes in adipokines levels in postmenopausal women [44] might be considered as risk factors of metabolic syndrome and its components. Moreover, we did not have information on the exact timings of dinner, bedtime, and the intake of dinners and snacks including alcohol. Future studies should assess factors such as sleep duration, menopause, and food intake carefully.

The main strength of this study is that we analyzed the associations between night eating habits and the prevalence metabolic syndrome and its components using a large-scale dataset derived from middle-aged men and women. Our data will help improve the quality of meal guidance, such as how to adopt a nutritional diet and how to improve eating environments.

## Conclusions

Having both night eating habits, “dinner immediately before bed” and “snacks after dinner”, was associated with higher BMI in both Japanese men and women. Men with both habits showed higher odds of dyslipidemia when compared to those with neither habit. Women with both habits had higher odds of metabolic syndrome and abdominal obesity when compared to those with neither habit. In men and women, both night eating habits were associated with dyslipidemia. The presence of both habits tended to show a supra-additive interaction on developing obesity in women. Hence, middle-aged individuals should be advised to refrain from both night eating habits to reduce the risk of developing complications associated with metabolic syndrome.

## Abbreviations

BMI: Body mass index
CI: Confidence interval
HbA1c: Glycated hemoglobin
HDL: High-density lipoprotein
LDL: Low-density lipoprotein
OR: Odds ratio
RERI: Relative excess risk due to interaction
VLDL: Very low-density lipoprotein
